# Application of Positive Matrix Factorization in the Identification of the Sources of PM_2.5_ in Taipei City

**DOI:** 10.3390/ijerph15071305

**Published:** 2018-06-21

**Authors:** Wen-Yuan Ho, Kuo-Hsin Tseng, Ming-Lone Liou, Chang-Chuan Chan, Chia-hung Wang

**Affiliations:** 1 Department of Environmental Protection, Taipei City Government, 6 Floor, No. 1, City Hall Road, Taipei 110, Taiwan; la-khtseng@mail.taipei.gov.tw (K.-H.T.); mingloneliou@gmail.com (M.-L.L.); 2 College of Public Health, National Taiwan University, No. 17, Xu-Zhou Road, Taipei 100, Taiwan; ccchan@ntu.edu.tw; 3 Sinotech Engineering Services, Ltd., 12 Floor, No. 171, Section 5, Nanjing E. Road, Songshan District, Taipei 105, Taiwan; vcnt@mail.sinotech.com.tw

**Keywords:** PM_2.5_, online monitoring, vertical profile, photochemical reaction, PMF

## Abstract

Fine particulate matter (PM_2.5_) has a small particle size, which allows it to directly enter the respiratory mucosa and reach the alveoli and even the blood. Many countries are already aware of the adverse effects of PM_2.5_, and determination of the sources of PM_2.5_ is a critical step in reducing its concentration to protect public health. This study monitored PM_2.5_ in the summer (during the southwest monsoon season) of 2017. Three online monitoring systems were used to continuously collect hourly concentrations of key chemical components of PM_2.5_, including anions, cations, carbon, heavy metals, and precursor gases, for 24 h per day. The sum of the concentrations of each compound obtained from the online monitoring systems is similar to the actual PM_2.5_ concentration (98.75%). This result suggests that the on-line monitoring system of this study covers relatively complete chemical compounds. Positive matrix factorization (PMF) was adopted to explore and examine the proportion of each source that contributed to the total PM_2.5_ concentration. According to the source contribution analysis, 55% of PM_2.5_ can be attributed to local pollutant sources, and the remaining 45% can be attributed to pollutants emitted outside Taipei City. During the high-PM_2.5_-concentration (episode) period, the pollutant conversion rates were higher than usual due to the occurrence of vigorous photochemical reactions. Moreover, once pollutants are emitted by external stationary pollutant sources, they move with pollution air masses and undergo photochemical reactions, resulting in increases in the secondary pollutant concentrations of PM_2.5_. The vertical monitoring data indicate that there is a significant increase in PM_2.5_ concentration at high altitudes. High-altitude PM_2.5_ will descend to the ground and thereby affect the ground-level PM_2.5_ concentration.

## 1. Introduction

Particulate matter (PM) refers to solid particles or droplets suspended in the atmosphere, and these often carry dioxin, heavy metals, and other harmful substances. PM can be inhaled into the body and accumulate in the trachea or lungs [1,2]. Therefore, inhalable PM has a greater effect on human health than all other air pollutants. PM is classified by its aerodynamic diameter. Coarse PM (e.g., PM_10_) reaches the ground within a few hours due to gravity. In contrast, fine PM (e.g., PM_2.5_) tends to remain suspended in the air for a long time and can thus be transported long distances, and, during their transport, photochemical reactions can occur [3].

PM_2.5_ has a wide variety of sources and is composed of many different chemical compounds. Due to its small particle size, PM_2.5_ can enter the respiratory mucosa and directly penetrate the alveoli and even the blood [4,5]. In 2013, the World Health Organization (WHO) listed PM_2.5_ as a major environmental carcinogen [6]. Short-term exposure to PM_2.5_ can increase respiratory tract allergies and related diseases and increases the risk of emergency ambulance dispatches for all-cause, respiratory, and neuropsychological reasons [7]. Long-term inhalation of PM_2.5_ might lead to heavy-metal poisoning, which is associated with chronic pulmonary and blood carcinomas. The mixing of PM_2.5_ with organic pollutants results in a risk for infertility because organic pollutants can affect the reproductive system and potentially increase the overall mortality rate [8,9].

An increasing number of countries have listed PM_2.5_ as a pollution indicator [10]. As early as 1999, the U.S. included 8-h ozone measurements and 24-h PM_2.5_ measurements in a new version of the Air Quality Index (AQI) [11,12]. Referring to the U.S. standards, Taiwan’s Environmental Protection Administration (EPA) also included PM_2.5_ in its air quality standards in May 2012: the standard annual average concentration was 15 μg/m^3^, and the standard daily average concentration was 35 μg/m^3^. According to PM_2.5_ data collected over the past 10 years (from 2007 to 2016 at a total of seven stations in the Taipei City area) by the Taipei City Government, the annual average PM_2.5_ concentration has improved steadily every year, from 32.5 μg/m^3^ in 2007 to 17.3 μg/m^3^ in 2016, which corresponds to a 46.8% improvement. Even though the various policies for pollution reduction are effective for gradually reducing the PM_2.5_ concentration each year, National Ambient Air Quality Standards have not yet been reached. Therefore, a series of more effective measurements must be implemented to reduce PM_2.5_ concentrations. A crucial step in more effectively reducing the PM_2.5_ concentration is to determine the sources of PM_2.5_ in the Taipei metropolitan area [13,14].

Studies analyzing the sources of atmospheric PM constitute the foundation, as well as a precondition, for PM prevention. In these studies, the most frequently adopted model is the receptor model [15], in which statistical pollutant information is analyzed together with PM_2.5_ concentrations from ambient observations [16]. Paatero and Tapper [17] proposed a technique called positive matrix factorization (PMF), which is a compositional analysis approach that has been widely used in recent years. This approach is particularly useful for regions for which there is insufficient pollution composition information [18,19,20]. A key feature of PMF is that it does not require the source composition as an input. Moreover, PMF allows users to use the standard deviation of the data for weighting, optimization, and handling missing data, and, as a result, this approach provides better flexibility and convenience for pollution source contribution analysis than the receptor model.

Air quality monitoring stations analyze only the hourly concentrations of PM_10_ and PM_2.5_, and such information provides only quantitative trends in the PM_10_ and PM_2.5_ concentrations for determining the level of pollution [21,22]. This analysis is not sufficient for determining the pollution sources of PM_2.5_. Thus, to investigate the sources contributing PM_2.5_ to Taipei City, this study used three online monitors to monitor the hourly changes in the PM_2.5_ composition, namely, the concentrations of anions, cations, carbon, heavy metals, and precursor gases of the major ions [23,24,25,26,27,28], and PMF was then adopted to estimate the ratios of the contributions from the different pollution sources.

## 2. Methodology

Aside from analyzing data collected by an existing air quality monitoring station (Chungcheng Air Quality Monitoring Station, 121°30′55′′ E, 25°2′10′′ N, The PM_2.5_ monitoring equipment is MET-ONE 1020; SO_2_ is THERMO 43i), this study also innovatively incorporated three online monitors for concurrent monitoring. The collected information was then combined with vertical PM_2.5_ observation data collected at different altitudes to analyze the sources of PM_2.5_ in the Taipei metropolitan area. Taipei City is the capital city and municipality of Taiwan. The city in Taiwan with the highest population density.

### 2.1. Monitoring Experiment

**Figure 1:**
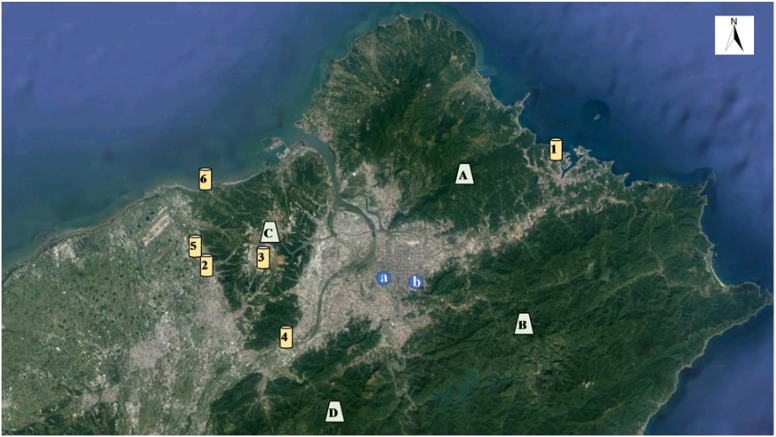
Environment surrounding the monitoring site: a indicates the Chungcheng Air Quality Monitoring Station, where online monitoring was also conducted, and b represents the vertical observation site at the Taipei 101 building. A shows the Tatung mountain group, B indicates the Songshan hills, C is the Linkou plateau, D indicates the Xueshan range, and 1–6 show the top six emission sources with the highest effects on the air quality in northern Taiwan.

The landforms of the monitoring site used in this study and the surrounding area are described below (Figure 1). Taipei City is located in the Taipei Basin in northern Taiwan. The Tatun Volcano Group (altitude between 800 m and 1200 m) is to the north, and the Linkou plateau (altitude between 240 and 250 m) and Guanyin mountain (altitude of 616 m) are located to the west. The Xueshan range is to the south, and the Songshan hills are to the southeast (the altitude of Xiangshan is 183 m). There are no industrial parks or large-scale factories in the Taipei City area, and most large-scale stationary pollution sources in this area are thus from the neighboring areas. For example, 89% of the SO_2_ emission level in 2016 originated from a large-scale oil fuel power station located northeast of Taipei City (the chimney is 200-m high), and the remaining pollution sources (11%) are distributed along the west side of the Taipei Basin.

Taiwan’s climate and weather are heavily influenced by monsoon; in summer, there is the southwest monsoon, while, in winter, there is the northeast monsoon. Taipei is typically affected by Asian Dust Storms every year from December to May of the following year [29,30]. To minimize influences from sandstorms, this investigation of PM_2.5_ pollution sources in the Taipei metropolitan area was conducted in the summer (from 8–30 August 2017), and samples were continuously collected 24 h for a total of 21 days.

**Figure 2:**
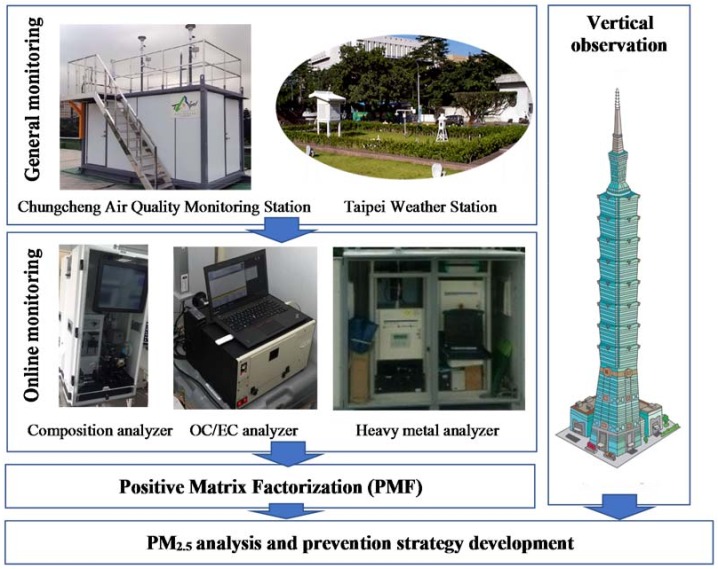
Monitoring infrastructure and data analysis methods.

The monitoring infrastructure and data analysis methods of this study is shown in Figure 2. The Chungcheng Air Quality Monitoring Station was set up by the Taipei Municipal Government’s Department of Environmental Protection and continuously monitors SO_2_, NO, NO_2_, O_3_, CO, PM_10_, PM_2.5_ and other air pollutants. This station also collaborates with a nearby Taipei Weather Station to monitor meteorological factors, including temperature, rainfall, and radiation. These stations are routinely maintained to ensure the accuracy and completeness of the monitoring data. To investigate the pollution sources, this study incorporated three online monitoring systems for the concurrent monitoring of the chemical composition of PM_2.5_. Using the changes in the hourly concentrations of various pollutants, the chemical composition of PM_2.5_ in Taipei City was analyzed, and this information was then used to determine the sources of PM_2.5_ in the Taipei metropolitan area. An in situ gas and aerosol composition monitor (Model: S-609EG) measured the concentrations of various aerosol ions (SO_4_^2−^, NO_3_^−^, Cl^−^, NH_4_^+^, Ca^2+^, Mg^2+^, K^+^, Na^+^ and other anions and cations) and many gaseous pollutants (HNO_2_, HNO_3_, and NH_3_). For the continuous monitoring of carbon fractions, this device uses the thermal-optical method to analyze the hourly concentrations of elemental carbon (EC) and organic carbon (OC). This approach is approved by the National Institute of Occupational Safety and Health (NIOSH) of the U.S. Tape-on-reel filter tape sampling and non-destructive X-ray fluorescence (XRF) (Model: Xact 625) analysis were adopted for the hourly monitoring of the concentrations of 21 atmospheric heavy metals: Ti (Titanium), V (Vanadium), Cr (Chromium), Mn (Manganese), Fe (iron), Co (Cobalt), Ni (Nickel), Cu (Copper), Zn (Zinc), Ga (Gallium), As (Arsenic), Se (Selenium), Mo (Molybdenum), Cd (Cadmium), Ba (Barium), Au (Gold), Hg (Mercury), Tl (Thallium) and Pb (Lead). All on-line monitoring equipment was operated by professional operators in accordance with standard operating procedures. After passing the test of the standard, the monitoring equipment performed the monitoring in the best condition. All monitoring equipment underwent a complete inspection and calibration procedure before the experiment.

To establish an approach for the vertical monitoring of PM_2.5_, this study took advantage of the height of Taipei 101 (which is approximately 509.2-m high, making it the eighth highest building in the world) by setting up cloud-based air quality monitors on the 6th, 50th, and 90th floors of Taipei 101 (which are located 40 m, 220 m, and 390 m aboveground, respectively). The long-term continuous monitoring of PM_2.5_ was established at various altitudes with the same time series, and the PM_2.5_ concentration was measured by a red laser and a semiconductor optical sensor with a resolution of 1 μg/m^3^.

### 2.2. PMF Model

The PMF model is a useful factor analysis method for estimating source profiles and contributions. The principle of PMF was described previously [17,19,20]. Briefly, the mathematical expression of PMF can be described as

(1) $$x_{ij}=\overset{P}{\underset{k=1}{\sum }}g_{ik}f_{kj}+e_{ij}$$

where *x_ij_* is the measured concentration of the *j*th species in the *i*th sample, *f_pj_* is the concentration of the *j*th species from the *p*th source, *g_ip_* is the contribution of the *p*th source to the *i*th sample, *e_ij_* is the portion of the measurements that cannot be fitted by the model (residuals), and *p* is the number of factors. PMF 5.0 was used in this study.

PMF provides a solution that minimizes an object function, *Q*, based upon the uncertainty for each observation [31], which is defined as:

(2) $$\;Q=\overset{n}{\underset{i=1}{\sum }}\overset{m}{\underset{j=1}{\sum }}{\left(\frac{e_{ij}}{s_{ij}}\right)}^{2}$$

(3) $$\;e_{ij}=x_{ij}-\overset{P}{\underset{k=1}{\sum }}g_{ik}f_{kj}$$

where *s_ij_* is the uncertainty in the measured data *x_ij_*. PMF uses a least-squares approach to solve the factor analysis problem with integrating non-negativity constraints into the optimization process, meaning that sources cannot have negative species concentration (*f_kj_* ≥ 0) and the sample cannot have a negative source contribution (*g_ki_* ≥ 0). The solution of Equation (2) is obtained using an iterative minimization algorithm, PMF [31]. PMF uses the error of measurement in the data to provide optimum data point scaling, and permits better treatment of missing and below detection-limit values. Measurement values, *x_ij_*, below the detection limit were replaced by a value of half of the detection limit, and an error corresponding to a relative uncertainty of 100% was assigned to the original error estimate.

## 3. Results and Discussion

### 3.1. Analysis of the Time Series of Monitoring Values

**Figure 3:**
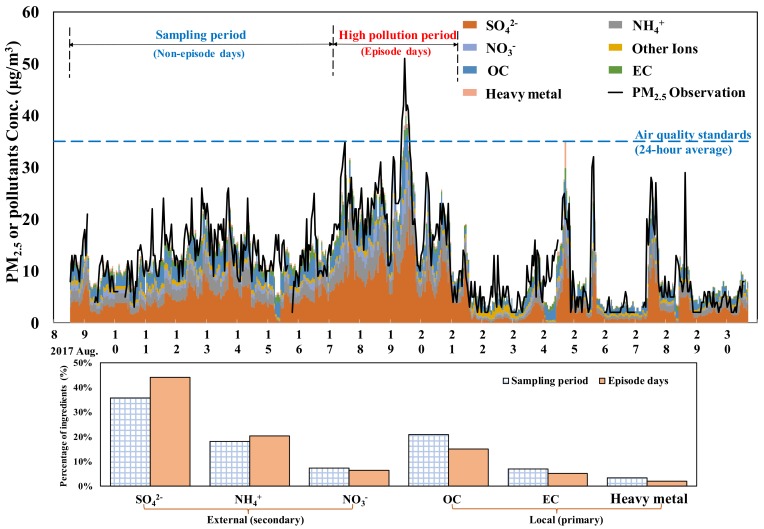
Time series of the concentrations of PM_2.5_ and its major chemical components. The other ions include Na^+^, K^+^, Mg^2+^, Ca^2+^, Cl^−^, and NO_2_^−^.

As shown in the literature, the primary chemical components of PM_2.5_ are SO_4_^2−^, NH_4_^+^, NO_3_^−^, OC, EC, heavy metals, and other anions and cations (including Na^+^, K^+^, Mg^2+^, Ca^2+^, Cl^−^, and NO_2_^−^) [31]. Figure 3 shows a chart of the hourly concentrations of the major chemical components over time (the sampling date is given on the x-axis, and the position of the number indicating the date is shown at the zero hour each day). The black solid line is the concentration trend curve of PM_2.5_ observed at Chungcheng Station, and the blue dotted line shows the daily air quality standard of PM_2.5_ (35 μg/m^3^). The colored blocks in the trend chart indicate the hourly distributions of various chemical components and the sums of their concentrations. The analysis showed that PM_2.5_ in the Taipei metropolitan area is mainly composed of SO_4_^2−^, NH_4_^+^, NO_3_^−^, OC, EC, heavy metals, and other anions and cations. This finding is consistent with the literature [32]. According to the time series chart, the sum of the data obtained from the three sets of online monitors is highly consistent with the PM_2.5_ concentration obtained from the monitoring station (coefficient of determination R^2^ = 0.73; an R^2^ of 1 indicates that the regression predictions perfectly fit the data).

During the monitoring period, the hourly concentration trend showed a steep change in the concentration of PM_2.5_ during 17–20 August and a relatively high concentration (the highest hourly concentration was detected on 19 August). Therefore, this study defined 17–20 August as a PM_2.5_ episode, and the data were compared with data collected on other sampling days (non-episode periods). Figure 3 shows the average percentages of main species (SO_4_^2^^−^, NH_4_^+^, NO_3_^−^, OC, EC, and heavy metals) on the episode and non-episode days. The proportions of primary pollutants on the episode days were lower than those during non-episode days, whereas the proportions of secondary pollutants, particularly SO_4_^2^^−^ and NH_4_^+^, were higher during episode days than during other days. The results showed that primary pollutants are emitted from external fixed sources of pollution, enter the Taipei metropolitan area with the air mass and undergo intense photochemical reactions during episode days.

The changes in the daily average concentrations of PM_2.5_ collected at Chungcheng Station and of the major chemical components collected in this study are summarized in Table 1. The average concentration of PM_2.5_ during the monitoring period (during 8–31 August, a total of 21 days) was 11.99 μg/m^3^. The highest value, 27.13 μg/m^3^, was observed on 19 August, and the lowest value, 2.63 μg/m^3^, was detected on 26 August. The chemical components analyzed by the three online monitoring systems were divided into seven categories. The analysis of the average concentration of each category showed that the highest concentration was obtained for SO_4_^2^^−^ (4.59 μg/m^3^, approximately 38.8%), followed by OC (19.7%), NH_4_^+^ (19.3%), NO_3_^−^ (6.7%), and EC (6.5%). The effect of heavy metals was relatively low at 2.7%. On the day with the highest daily average concentration of PM_2.5_ (19 August), the concentration of SO_4_^2^^−^ accounted for as much as 46% of the total PM_2.5_ concentration. However, the Taipei metropolitan area has no major source of SO_2_ pollution. The detected SO_2_ is presumably a product of photochemical reactions involving emissions from stationary pollution sources outside the metropolitan region that were transported to the metropolitan region by air masses. Furthermore, the sum of the daily average concentrations of all major species was 11.84 μg/m^3^, which suggests that the species analyzed in this study comprise approximately 98.75% of the actual mass concentration of PM_2.5_.

### 3.2. Automatic Continuous Hourly Monitoring: Daily Trends

This study analyzed PM_2.5_ in the Taipei metropolitan area, and the results presented in previous section showed that the total concentrations of SO_4_^2−^, NO_3_^−^, and NH_4_^+^ accounted for 64.7% of the chemical composition of PM_2.5_ (38.8%, 6.7%, and 19.3%, separately). These compounds are all secondary aerosol particles, which are produced by a series of highly complex chemical changes and photochemical reactions in the atmosphere. An analysis of the basic data obtained from the hourly monitoring of PM_2.5_ and its major chemical components is presented below.

(A) Fine particulate matter (PM_2.5_)

**Figure 4:**
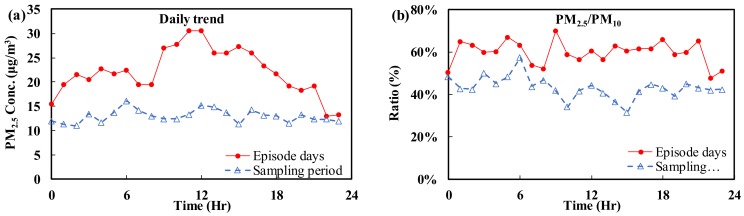
Hourly changes in air quality pollutants on episode days and during the sampling period: (**a**) PM_2.5_; and (**b**) PM_2.5_/PM_10_.

This study used PM_2.5_ data collected by the Taipei City Chungcheng Air Quality Monitoring Station to analyze the changes in the PM_2.5_ concentration at different time points over an entire day (Figure 4). The line shows the daily changes in PM_2.5_ measured at Chungcheng Station during episode and non-episode days. As shown in Figure 4a, during the PM_2.5_ episode, the concentration of PM_2.5_ started to increase between 6:00 and 7:00 each day, peaked at 12:00 and then started to decrease after 13:00. No significant trend in the concentration was found during the sampling period, and, moreover, the overall concentration during the non-episode period was lower than that during the episode days. This trend is similar to that found for O_3_. In addition to common pollution sources, photochemical reactions can also contribute to the PM_2.5_ concentration during the PM_2.5_ episode days. Figure 4b shows the ratio of the PM_2.5_ (fine) concentration to the PM_10_ (coarse) concentration, and the daily hourly changes did not reveal a significant trend. However, the average PM_2.5_/PM_10_ ratio during the episode days is 60%, which is higher than that during the sampling period (30%). This finding shows that the suspended particle content in the atmosphere during the episode days was increased mainly due to the presence of PM_2.5_.

(B) Sulfur oxides (SO_2_, SO_4_^2−^)

As shown in Table 1, the total SO_4_^2−^ concentration accounts for 38.8% of the chemical composition of PM_2.5_. SO_4_^2−^ is produced from SO_2_ through either a gas-phase homogeneous reaction or a liquid-phase heterogeneous reaction (e.g., Equation (4)). SO_2_ is mainly produced by the combustion of fossil fuels at places such as large-scale fuel power plants.

(4) $$SO_{2}\to H_{2}SO_{4}\to SO_{4}^{2-}$$

**Figure 5:**
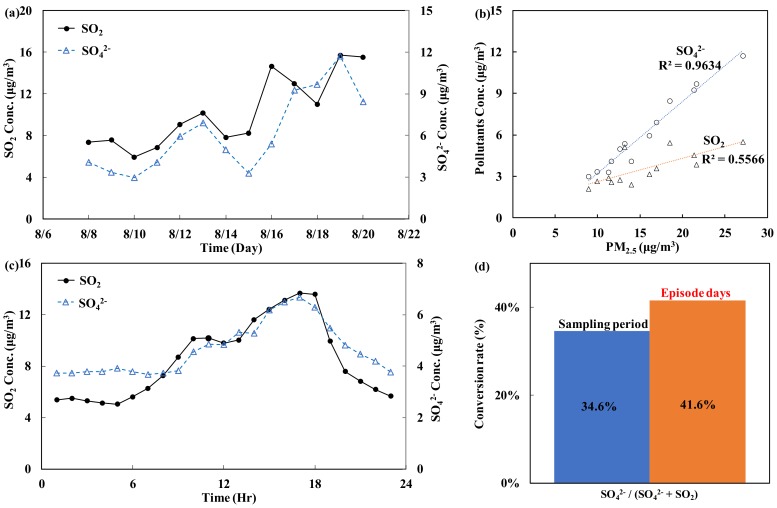
Trend in the daily and hourly average concentrations and conversion analysis of sulfur oxides: (**a**) daily average trend; (**b**) correlation with PM_2.5_; (**c**) hourly average trend; and (**d**) conversion rate.

Figure 5a,b shows the correlation between the PM_2.5_ concentration and the SO_2_/SO_4_^2−^ ratio during the monitoring period. The results indicate that the levels of PM_2.5_ and SO_4_^2−^, one of the chemical components of PM_2.5_, are highly correlated, with an R^2^ value of 0.96 (*p* < 0.05). The levels of PM_2.5_ and the precursor SO_2_ are less correlated (R^2^ = 0.56, *p* < 0.05). Figure 5c shows the distribution of the daily hourly average concentrations of SO_2_ (the source) and SO_4_^2−^ (the product) during the monitoring period. The trend shows that increases in the concentration of SO_2_ are associated with increases in the concentration of SO_4_^2−^.

If all SO_4_^2−^ in the atmosphere originates only from SO_2_, increased SO_2_ conversion will lead to a higher concentration of SO_4_^2−^. Figure 5d shows the conversion rates of SO_2_/SO_4_^2−^ on an episode day and a non-episode day. The results suggest that the conversion rate of SO_2_/SO_4_^2−^ on the episode days is 41.6%, which is higher than that during the sampling period (34.6%). This finding indicates that the environmental and climate factors (poor dispersion) on episode days favor the conversion of SO_2_ to SO_4_^2−^ and thereby increase the PM_2.5_ concentration.

(C) NH_3_/NH_4_^+^

The precursor of NH_4_^+^ is NH_3_, which is a key species for the neutralization of acidic components in the atmosphere. During neutralization, NH_4_^+^ can form microparticles (e.g., Equation (5)).

*NH*_3_ + *Acidic components* → *NH*_4_^+^ (5)

**Figure 6:**
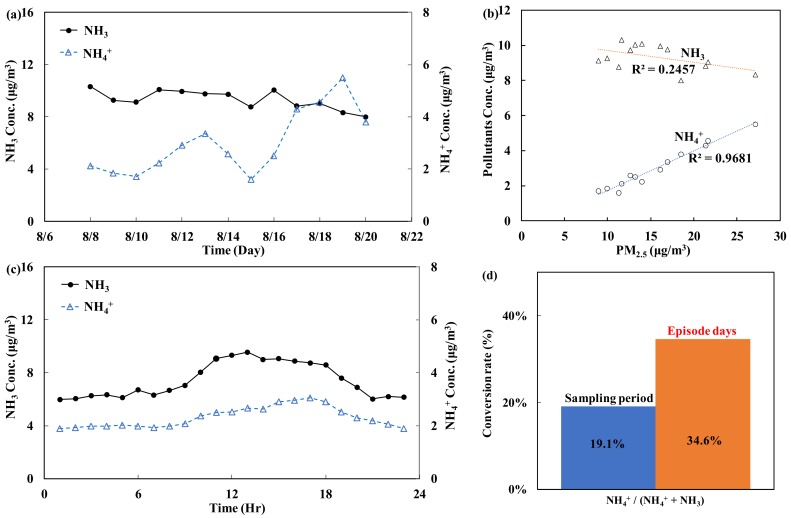
Trends in the daily and hourly average concentrations and conversion analysis of NH_3_/NH_4_^+^: (**a**) daily average trend; (**b**) correlation with PM_2.5_; (**c**) hourly average trend; and (**d**) conversion rate.

Figure 6a,b shows the correlation between the concentration of PM_2.5_ and NH_3_/NH_4_^+^ during the monitoring period. The results suggest that the levels of PM_2.5_ and NH_4_^+^, one of the chemical components (reactants) of PM_2.5_, are highly correlated, with an R^2^ value of 0.97 (*p* < 0.05). However, the levels of PM_2.5_ and the precursor NH_3_ are less correlated (R^2^ = 0.25, *p* < 0.05). Figure 6c shows the distribution of the daily hourly average concentrations of NH_3_ (the source) and NH_4_^+^ (the product) during the monitoring period. The changes show that the concentration of NH_3_ started to increase at 9:00, peaked at 12:00 and then started to decline at 18:00. As the concentration of NH_3_ increased, the concentration of NH_4_^+^ also increased, reaching a peak at 18:00.

If all NH_4_^+^ in the atmosphere originates from NH_3_, an increased conversion rate will lead to a higher concentration of NH_4_^+^. Figure 6d shows the rate of the conversion of NH_3_ to NH_4_^+^ on the episode days and the sampling period. The results suggest that the conversion rate of NH_3_ to NH_4_^+^ on the episode days was 34.6%, which is higher than the value of 19.1% observed during the sampling period. This finding indicates that environmental and climate factors (poor dispersion) on episode days favor the conversion of NH_3_ to NH_4_^+^, which in turn increases the PM_2.5_ concentration.

(D) Carbon (EC/OC)

**Figure 7:**
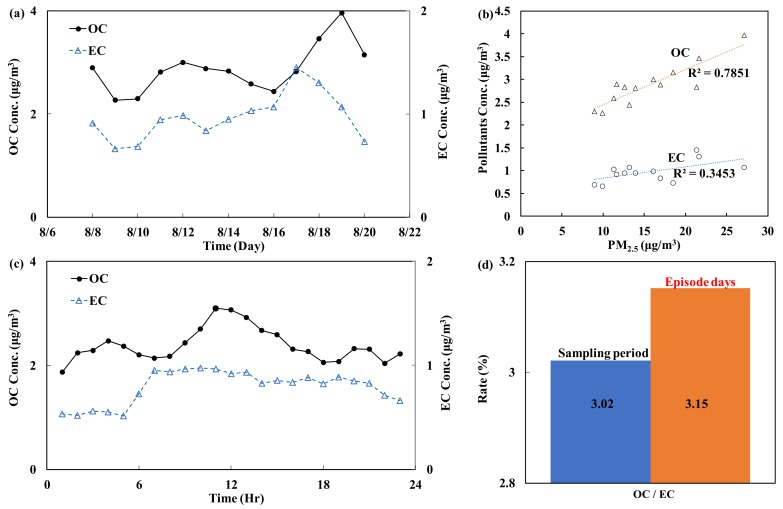
Carbon concentrations during the monitoring period: (**a**) daily average trend; (**b**) correlations between OC and PM_2.5_ and between EC and PM_2.5_; (**c**) hourly average trend; and (**d**) OC/EC ratio.

Carbon compounds (OC and EC) are emitted from incomplete combustion of fossil fuels. According to the analysis of the PM_2.5_ chemical composition in this study, OC accounts for 19.7% of PM_2.5_, while EC accounts for only 6.5%. Figure 7a,b shows the correlation between PM_2.5_ and OC/EC. The daily average concentration of OC was highly correlated with the PM_2.5_ concentration (R^2^ = 0.79, *p* < 0.05). Figure 7c shows the daily hourly changes in carbon fractions (OC and EC) during the monitoring period. The OC concentration was significantly higher than the EC concentration, and the daily hourly changes in OC were more apparent than those in EC. The OC concentration started to increase after 7:00, peaked at 12:00 and then declined, and the lowest OC concentration was detected in the evening. In contrast, the daily changes in the EC concentration did not show an apparent trend.

Furthermore, studies have shown that the OC/EC ratio can be used as a rough indicator of the pollution source; a ratio greater than 3 [25,26,33] indicates that vehicle emissions provide a relatively high contribution to the PM_2.5_ concentration. Figure 7c also shows that OC accounted for a greater proportion of PM_2.5_ than EC. The OC/EC ratios during episode days were higher than those during the sampling period (Figure 7d). This finding indicates that vehicle emissions make a relatively high contribution to the PM_2.5_ concentration during episode days.

### 3.3. PM Pollution Source Classification

This study used PMF to analyze the chemical composition of PM_2.5_ in the Taipei metropolitan area. The researchers screened the characteristic species in the factor profile to determine possible pollution sources of PM_2.5_ measured at the monitoring stations. First, the IM/IS method (“IM” is the maximum individual column mean, and “IS” is the maximum individual column standard deviation) [34,35] was adopted to determine the most suitable number of pollution source factors. The analysis considering six factors revealed that both IM and IS showed a significant trend. Therefore, the cases monitored in this study can be divided into six factor profiles.

**Figure 8:**
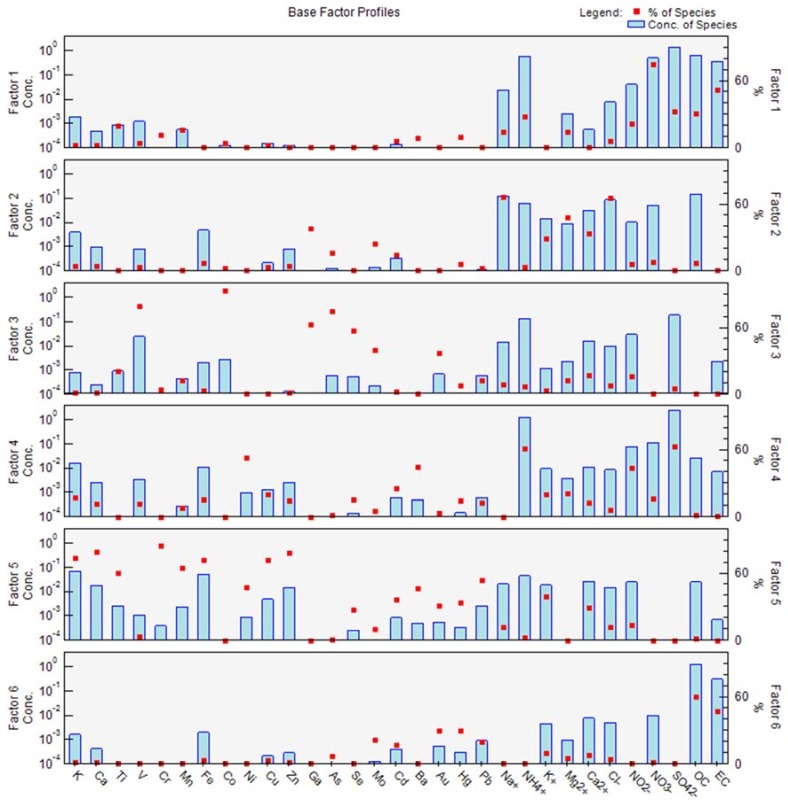
PMF analysis of the distribution of PM_2.5_ emission sources during the monitoring period (units: μg/m^3^).

The PMF modeling results identified six source factors. The factors were named according to the chemical component accounting for the highest fraction of the factor profile. The results from the analysis of the major characteristic species of each factor are presented in Table 2 and Figure 8. These major characteristic species and the possible corresponding emission sources are discussed below.

Factor 1 This factor includes four major characteristic species: NO_3_^−^, EC, SO_4_^2−^ and OC. The major source of EC and NO_3_^−^ is vehicles, particularly exhaust emitted by vehicles with a diesel engine.
Factor 2 This factor is composed of four major characteristic species: Na^+^, Cl^−^, Mg^2+^ and Ca^2+^. These major characteristic species can be classified as originating from sea spray.
Factor 3 The major characteristic species included in this factor are Co, V, As, Ga, and Se, which are fuel indicators. As, Ga, and Se might also be coal indicators. After integrating the major characteristic species of this factor, the emissions can be attributed to oil boilers used by hospitals, hotels and restaurants in the city [36,37].
Factor 4 This factor incorporates the following major characteristic species: NH_4_^+^, SO_4_^2−^, Ni, and Ba. These species are mainly derived from pollutants emitted by industrial entities. One example is pollutants transported by air masses, which undergo photochemical reactions and attach to PM. These types of pollutants might be PM_2.5_ derived from large-scale industrial sources (e.g., power plants and petrochemical plants) at locations upwind of the metropolitan area.
Factor 5 The major characteristic species incorporated in this factor are Cr, Ca, Zn, Cu, Fe, and Mn. These elements originate from the Earth’s crust. Fe and Mn are indicator species of dust and are mainly derived from street dust.
Factor 6 The major characteristic species incorporated in this factor are OC, OC, Au, Hg, and Pb. According to the VOC emission inventory in Taipei, gasoline vehicles and motorcycles are the major source of these species. Because the sampling site is near a road, OC might originate from the exhaust of gasoline vehicles, including motorcycles [38,39,40].

Table 2 shows the analysis results. Factor 4 makes the greatest contribution (40%) to the PM_2.5_ concentration. The major characteristic pollutant in this factor is SO_4_^2−^, which is related to emissions transported from large-scale pollution sources. As mentioned previously, the Taipei metropolitan area has no significant SO_2_ emission sources (power plant, oil refinery, etc.). SO_2_ is presumably emitted by large-scale stationary pollution sources around the metropolitan area and is transported by air masses. In addition, SO_4_^2−^ is thought to be generated from photochemical reactions. This mechanism explains how SO_2_ contributes to the PM_2.5_ concentration in the Taipei metropolitan area. Factor 1 makes the second highest contribution. According to its major characteristic species, this factor originates from emissions from diesel-powered vehicles and accounts for 32.8% of the total PM_2.5_. Vehicle exhaust emissions (Factor 6) make the third largest contribution to the PM_2.5_ concentration. The six factors identified in this study can be divided into two groups according to their emission sources and characteristics: local pollution sources and foreign pollution sources. Factors 1, 3, 5, and 6 are related to locally generated pollution sources, whereas Factors 2 and 4 come from pollutant sources outside the Taipei metropolis area. Approximately 55% of the PM_2.5_ in the Taipei metropolitan area can be attributed to local pollution sources, whereas 45% of the PM_2.5_ originates from foreign pollution sources.

### 3.4. Vertical PM_2.5_ Concentration Trends

**Figure 9:**
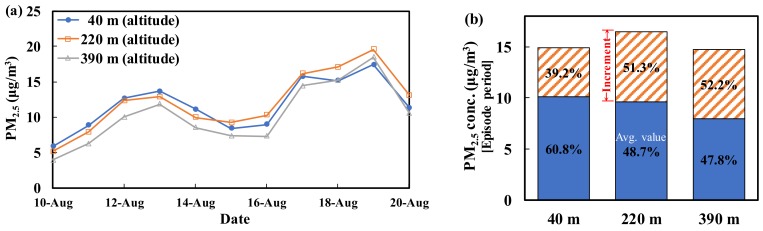
Mean PM_2.5_ concentrations measured at different altitudes at Taipei 101 during 10—20 August. (**a**) Daily mean, (**b**) Incremental analysis.

This study measured the PM_2.5_ mass concentration at 40 m, 220 m, and 390 m above the ground. The PM_2.5_ concentrations measured at the three high-altitude points at Taipei 101 used for monitoring were highly correlated with the PM_2.5_ concentration measured at Chungcheng Station. The coefficients of determination (R^2^) for the correlations at 40 m, 220 m, and 390 m were 0.88, 0.94, and 0.94 (*p* < 0.05), respectively. This finding indicates that the data collected at Taipei 101 at different altitudes consistently matched the PM_2.5_ levels measured at the ground. Therefore, high-altitude concentrations can be used for the analysis of PM_2.5_ sources. Figure 9 shows the changes in the daily average PM_2.5_ concentration measured at different altitudes at Taipei 101 during 10–20 August. The PM_2.5_ concentrations measured at 390 m (the highest altitude in this study) were always lower than those detected at the other tested altitudes, whereas the values measured at 40 m and 220 m varied depending on the pollution sources and the weather conditions. Figure 9 also shows the average PM_2.5_ concentrations at the three elevations during the episode period and the sampling period. During the episode period (18–20 August), the PM_2.5_ concentrations measured at 220 m were greater than those measured at 40 m, which is contrary to the trend observed during the sampling period. This phenomenon suggests that the increase in the PM_2.5_ concentration at high altitude is greater (51.3% for 220 m and 52.2% for 390 m) than that on the ground (39.2% for 40 m). Thus, the high-altitude PM_2.5_ will descend to the ground, affecting the value measured there.

**Figure 10:**
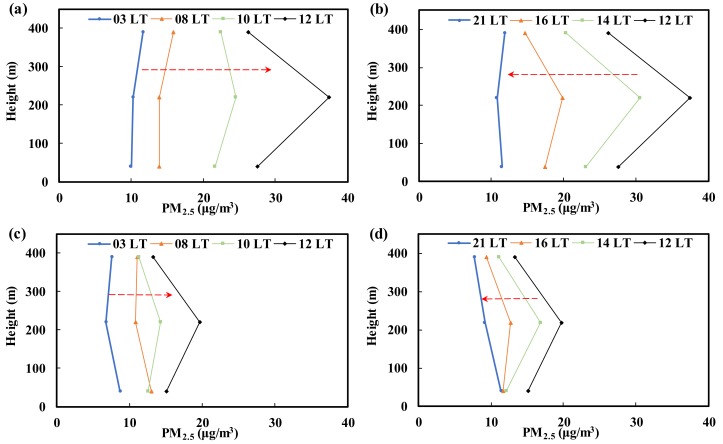
Analysis of the PM_2.5_ vertical concentration profiles at different time points. (**a**,**b**) Episode days; (**c**,**d**) Sampling period.

PM_2.5_ easily floats in the atmosphere and moves with the contaminated air mass. Nonetheless, due to either increased mass resulting from particle growth during transport or a lack of suitable weather conditions for transport (e.g., wind speed slowing down), the particles reach the ground based on their own weight. This study took advantage of the height of Taipei 101 to simultaneously collect PM_2.5_ samples at various heights at the same time series. These samples were then used to establish the vertical profiles of PM_2.5_ in the Taipei metropolitan area. Figure 10 shows the vertical profiles of PM_2.5_ established using data collected from the three different heights. The times 3:00 and 21:00 represent the night profile, and because human activities are reduced at that period and no photochemical reactions occur, this profile can be considered the background. During the daytime, a profile was established every two hours (8:00, 10:00, 12:00, 14:00, and 16:00). When photochemical reactions are induced by sunlight and the landform and wind flow are suitable, air masses with a high concentration of pollutants can be transported along the Songshan hills (the altitude of Xiangshan is 183 m) to the Taipei metropolitan area. If there is sufficient energy from sunlight and an adequate reaction time during this transport, various PM_2.5_ chemical components will be produced. These reactions will speed up the production of PM_2.5_ at 220 m, which would allow the peak concentration to be reached. The PM_2.5_ concentration at 220 m was higher than the concentration on the ground, but due to the sedimentation effect, the PM_2.5_ concentration on the ground will also eventually increase.

Figure 10 shows the PM_2.5_ vertical profiles at different time points on episode days. According to the figure, one can assume that atmospheric photochemical reactions will occur in the presence of sunlight, resulting in an increase in the PM_2.5_ concentration at high altitude. As a result, the entire profile shifts toward the right as time progresses. The peak concentration was reached at 12:00, and the increase in the PM_2.5_ concentration was most significant at 220 m (the increase was approximately 35 μg/m^3^). In the afternoon, sunlight and the resulting photochemical reactions were reduced. Therefore, the PM_2.5_ concentration decreases, and the entire profile moves toward the left over time (Figure 10b). According to these findings, it can be assumed that photochemical reactions provide a significant contribution to PM_2.5_ production.

Based on the monitoring and PMF analysis results, atmospheric SO_4_^2−^ was found to make the greatest contribution to the PM_2.5_ concentration in this study. This type of derivative stationary pollutant is generated from chemical reactions. Transportation vehicles and boilers in the metropolitan area emit limited amounts of SO_2_, which do not significantly increase the concentration of PM_2.5_. According to Figure 1, there is a northeast–southwest gap from Keelung to Taipei, and the Hischih Air Quality Monitoring Station is located in this route. If SO_2_ emitted by the Taipower plant (the emission quantity accounts for approximately 89%) is transported to the Taipei metropolitan area through this route, the synergistic effect of climate and light will induce the occurrence of photochemical reactions during transport, making SO_2_ the major source of SO_4_^2−^ in the Taipei metropolitan area, and the produced SO_4_^2−^ will increase the concentration of PM_2.5_.

**Figure 11:**
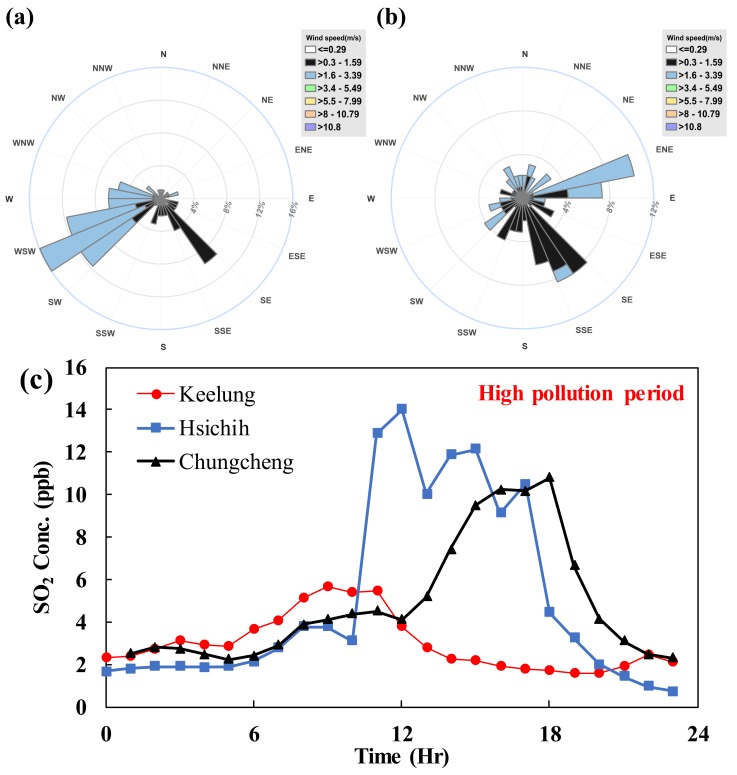
Transport diagram. Wind field during (**a**) Sampling period, (**b**) Episode days, and (**c**) SO_2_ at different locations.

The wind speed and direction at Chungcheng Station were analyzed and plotted in wind rose charts for the episode days and sampling period (see Figure 11). Figure 11a shows the wind field characteristics during the sampling period, and the results suggest a normal summer monsoon pattern, i.e., with a prevailing southwest wind. On episode days, the wind field was less affected by the monsoon, the east wind prevailed, and the wind speed was high (Figure 11b). An analysis of the wind flow on episode days indicates that the polluted air masses presumably arrive at the metropolitan area through the route at the northeast side of the metropolitan area. Figure 11c shows the hourly SO_2_ concentration collected on episode days at each air quality measuring station, which are located on the air mass transport route. The location of each air quality monitoring station and the trends in the SO_2_ concentration indicate that the pollution air masses emitted by the large-scale power plant are transported to the Taipei metropolitan area through the opening of the basin at the northeast side. Due to the topography of the transmission path, the PM_2.5_ concentration at high altitude shows the first effects after the air mass enters the Taipei Basin.

## 4. Conclusions

This study used three online monitoring systems to monitor the hourly concentration of PM_2.5_ and its main chemical components, including anions, cations, carbon fractions, heavy metals, and precursor gases, in Taipei City. The monitoring results showed that the major chemical components of PM_2.5_ are predominantly derivative species, with the highest contribution made by SO_4_^2−^, followed by OC and NH_4_^+^. The total concentrations of these three derivative species accounted for 64.7% of the chemical composition of PM_2.5_ (38.8%, 6.7%, and 19.3%, separately). During the high-PM_2.5_-pollution period, the increase in the PM concentration is mainly due to the increase in the proportion of PM_2.5_ (from 40% to 60%), and the effect of photochemical reactions is significant (conversion rates were higher).

The study used PMF to analyze the proportion of PM_2.5_ pollution source contributions by screening the characteristic species of the factor profile. The Taipei metropolis area is a commercial and financial city, and there are no large-scale emission sources (power plant, oil refinery, industrial area, etc.) in this area. The six factors identified in the PMF analysis can be divided into two groups according to their emission sources and characteristics: local pollution sources (vehicles and boilers) and pollution sources outside Taipei City. The results showed that 55% of the PM_2.5_ is derived from local pollutants and that the remaining 45% originates from pollutant sources outside the Taipei metropolitan area.

This study also measured the PM_2.5_ mass concentration at 40 m, 220 m, and 390 m above the ground to establish vertical profiles of PM_2.5_. The PM_2.5_ concentrations measured at 390 m were always lower than those measured at the other altitudes, whereas the values measured at 40 m and 220 m varied depending on the pollution sources and the weather conditions. During the episode days, the peak concentration was reached at 12:00, and the increase in the PM_2.5_ concentration was most significant at 220 m due to stable atmospheric conditions. In the afternoon, sunlight and the resulting photochemical reactions weaken, and the PM_2.5_ concentration therefore decreases. Therefore, photochemical reactions significantly contribute to PM_2.5_ production. The vertical profile analysis also indicated that air pollutants outside the city were transported through the Taipei Basin on episode days. To achieve the goal of reducing the PM_2.5_ concentration, more attention needs to be paid to cross-border governance in addition to reducing local pollution sources.

## Figures and Tables

**Table 1:** Analysis of the daily average concentrations of the chemical components of PM_2.5_ during the monitoring period (units: μg/m^3^).

Date	OC	EC	SO_4_^2−^	NH_4_^+^	NO_3_^−^	Other Anions	Heavy Metals	All Chemical Components	PM_2.5_
8 August	2.90	0.91	4.09	2.12	1.04	0.86	0.55	12.47	11.64
9 August	2.27	0.66	3.35	1.83	0.81	0.75	0.47	10.14	9.95
10 August	2.30	0.69	2.98	1.71	0.73	0.67	0.39	9.46	8.95
11 August	2.81	0.94	4.08	2.23	0.96	0.90	0.43	12.36	13.96
12 August	3.00	0.99	5.93	2.92	1.16	0.93	0.48	15.40	16.13
13 August	2.88	0.84	6.92	3.36	1.17	0.93	0.43	16.52	16.96
14 August	2.83	0.95	4.96	2.59	1.02	0.80	0.42	13.57	12.63
15 August	2.58	1.03	3.28	1.60	0.67	0.66	0.39	10.22	11.29
16 August	2.44	1.07	5.37	2.50	0.99	0.70	0.37	13.44	13.21
17 August	2.83	1.45	9.25	4.29	1.45	0.87	0.44	20.58	21.38
18 August	3.46	1.30	9.68	4.55	1.51	0.87	0.51	21.89	21.67
19 August	3.96	1.07	11.72	5.49	1.91	0.85	0.49	25.50	27.13
20 August	3.15	0.73	8.44	3.80	0.88	0.69	0.40	18.10	18.50
21 August	1.29	0.51	3.68	1.71	0.47	0.93	0.14	8.73	6.54
22 August	1.10	0.26	0.62	0.48	0.28	1.33	0.07	4.14	5.25
23 August	1.62	0.70	1.78	0.96	0.62	0.69	0.12	6.50	6.75
24 August	2.74	1.06	4.35	2.06	1.04	0.68	0.50	12.42	13.41
25 August	2.04	0.62	2.81	1.47	0.06	0.55	0.16	7.72	8.50
26 August	1.29	0.19	0.73	0.55	0.02	0.45	0.10	3.33	2.63
27 August	2.21	0.50	4.23	2.16	0.06	0.54	0.19	9.90	13.22
28 August	1.69	0.43	2.90	1.44	0.50	0.54	0.21	7.70	7.63
29 August	1.11	0.36	1.97	1.22	0.35	0.51	0.09	5.61	3.96
30 August	1.06	0.37	2.45	1.50	0.40	0.70	0.09	6.56	4.43
Mean	2.33	0.77	4.59	2.28	0.79	0.76	0.32	11.84	11.99
Percent	19.7%	6.5%	38.8%	19.3%	6.7%	6.4%	2.7%	100% (98.75%)	

**Table 2:** PMF analysis of major characteristic species.

Factor	Major Characteristic Species	Possible Emission Sources	Contribution Percentage (%)
Factor 1	NO_3_^−^, EC, SO_4_^2−^, OC	Diesel vehicle exhaust Exhaust emissions	32.8%
Factor 2	Na^+^, Cl^−^, Mg^2+^, Ca^2+^	Sea salt spray	5.0%
Factor 3	Co, V, As, Ga, Se	Boiler combustion	3.9%
Factor 4	NH_4_^+^, SO_4_^2−^, Ni, Ba	NH_4_^+^, NO_3_^−^ Emissions transported from large-scale pollution sources	40.0%
Factor 5	Cr, Ca, Zn, Cu, Fe, Mn	Crustal elements, street dust	3.2%
Factor 6	OC, EC, Au, Hg, Pb	Gasoline vehicle exhaust	15.1%
Total			100.0%

## References

[1] Lo W.C., Shie R.H., Chan C.C., Lin H.H. (2017). Burden of disease attributable to ambient fine particulate matter exposure in Taiwan. J. Formos. Med. Assoc..

[2] Zhang Y.L., Cao F. (2015). Fine particulate matter (PM_2.5_) in China at a city level. Sci. Rep..

[3] Joseph A.E., Unnikrishnan S., Kumar R. (2011). Chemical Characterization and Mass Closure of Fine Aerosol for Different Land Use Patterns in Mumbai City. Ambient Air Qual. Res..

[4] Apte J.S., Marshall J.D., Cohen A.J., Brauer M. (2015). Addressing Global Mortality from Ambient PM_2.5_. Environ. Sci. Technol..

[5] Chan C., Yao X. (2008). Air pollution in mega cities in China. Atmos. Environ..

[6] World Health Organization (WHO) (2013). Health Risks of Air Pollution in Europe—HRAPIE Project: Recommendations for Concentration—Response Functions for Cost-Benefit Analysis of Particulate Matter, Ozone and Nitrogen Dioxide.

[7] Phung V.L.H., Ueda K., Kasaoka S., Seposo X., Tasmin S., Yonemochi S., Phosri A., Honda A., Takano H., Michikawa T. (2018). Acute Effects of Ambient PM_2.5_ on All-Cause and Cause-Specific Emergency Ambulance Dispatches in Japan. Int. J. Environ. Res. Public Health.

[8] Wan Mahiyuddin W.R., Sahani M., Aripin R., Latif M.T., Thach T.Q., Wong C.M. (2013). Short-term effects of daily air pollution on mortality. Atmos. Environ..

[9] European Environment Agency (EEA) (2014). Air Quality in Europe—2014 Report.

[10] United Nations (UN) (2014). Open Working Group on Sustainable Development Goals. http://sustainabledevelopment.un.org.

[11] European Environment Agency (EEA) (2013). Air Quality in Europe—2013 Report.

[12] Linares C., Díaz J. (2009). Impact of particulate matter with diameter of less than 2.5 microns (PM_2.5_) on daily hospital admissions in 0~10 year olds in Madrid, Spain (2003–2005). Gac. Sanit..

[13] Zhang R., Jing J., Tao J., Hsu S., Wang G., Cao J., Les C., Zhu L., Chen Z., Zhao Y. (2013). Chemical characterization and source apportionment of PM_2.5_ in Beijing: Seasonal perspective. Atmos. Chem. Phys..

[14] Kim E., Turkiewicz K., Zulawnick S.A. (2010). Sources of fine particles in the South Coast area, California. Atmos. Environ..

[15] Pant P., Harrison R.M. (2012). Critical review of receptor modelling for particulate matter: A case study of India. Atmos. Environ..

[16] Hopke P.K. (1985). Receptor Modeling in Environmental Chemistry.

[17] Paatero P., Tapper U. (1994). Positive matrix factorization: A non-negative factor model with optimal utilization of error estimates of data values. Environmetrics.

[18] Tiwari S., Pervez S., Perrino C., Bisht D.S., Srivastava A.K., Chate D. (2013). Chemical characterization of atmospheric particulate matter in Delhi, India, Part II: Source apportionment studies using PMF 3.0. Atmos. Res..

[19] Amato F., Polfi M., Escrig A., Querol X., Alastuey A., Pey J., Perez N., Hopke P.K. (2009). Quantifying road dust resuspension in urban environment by multilinear engine: A comparison with PMF2. Atmos. Environ..

[20] Amato F., Hopke P.K. (2012). Source apportionment of the ambient PM_2.5_ across St. Louis using constrained positive matrix factorisation. Atmos. Environ..

[21] Zhao Q., Shen G., Li L., Chen F., Qiao Y., Li C., Liu Q., Han J. (2017). Ambient Particles (PM_10_, PM_2.5_ and PM_1.0_) and PM_2.5_ Chemical Components in Western Yangtze River Delta (YRD): An Overview of Data from 1-year Online Continuous Monitoring at Nanjing. Aerosol Sci. Eng..

[22] Gao M., Cao J., Seto E. (2015). A distributed network of low-cost continuous reading sensors to measure spatiotemporal variations of PM_2.5_ in Xi’an, China. Environ. Pollut..

[23] Ten Brink H., Otjes R., Jongejan P., Slanina S. (2007). An instrument for semi-continuous monitoring of the size-distribution of nitrate, ammonium, sulphate and chloride in aerosol. Atmos. Environ..

[24] Bigi A., Bianchi F., De Gennaro G., Di Gilio A., Fermo P., Ghermandi G., Prévôt A.S.H., Urbani M., Valli G., Vecchi R. (2017). Hourly composition of gas and particle phase pollutants at a central urban background site in Milan, Italy. Atmos. Res..

[25] Gao J., Peng X., Chen G., Xu J., Shi G.L., Zhang Y.C., Feng Y.C. (2016). Insights into the chemical characterization and sources of PM_2.5_ in Beijing at a 1-h time resolution. Sci. Total Environ..

[26] Vodička P., Schwarz J., Ždímal V. (2013). Analysis of one year’s OC/EC data at a Prague suburban site with 2-h time resolution. Atmos. Environ..

[27] Wang M., Shao M., Chen W., Yuan B., Lu S., Zhang Q., Zeng L., Wang Q. (2014). A temporally spatially resolved validation of emission inventories by measurements of ambient volatile organic compounds in Beijing, China. Atmos. Chem. Phys..

[28] Park S.S., Cho S.Y., Jo M.R., Gong B.J., Park J.S., Lee S.J. (2014). Field evaluation of a near-real time elemental monitor and identification of element sources observed at an air monitoring supersite in Korea. Atmos. Pollut. Res..

[29] Liang C.S., Yu T.Y., Lin W.Y. (2015). Source Apportionment of Submicron Particle Size Distribution and PM_2.5_ Composition during an Asian Dust Storm Period in Two Urban Atmospheres. Aerosol Air Qual. Res..

[30] Lai L.W. (2015). Fine particulate matter events associated with synoptic weather patterns, long-range transport paths and mixing height in the Taipei Basin, Taiwan. Atmos. Environ..

[31] Paatero P. (2002). User’s Guide for Positive Matrix Factorization Programs PMF2 and PMF3 Part 2: Reference.

[32] Chang S.C., Chou C.C.K., Chan C.C., Lee C.T. (2010). Temporal characteristics from continuous measurements of PM_2.5_ and speciation at the Taipei Aerosol Supersite from 2002 to 2008. Atmos. Environ..

[33] Sillanpää M., Frey A., Hillamo R., Pennanen A.S., Salonen R.O. (2005). Organic, elemental inorganic carbon in particulate matter of six urban environments in Europe. Atmos. Chem. Phys..

[34] Lee E., Chan C.K., Paatero P. (1999). Application of positive matrix factorization in source apportionment of particulate pollutants in Hong Kong. Atmos. Environ..

[35] Han J.S., Moon K.J., Lee S.J., Kim Y.J., Ryu S.Y., Cliff S.S., Yi S.M. (2006). Size-resolved source apportionment of ambient particles by positive matrix factorization at Gosan background site in East Asia. Atmos. Chem. Phys..

[36] Zhao M., Zhang Y., Ma W., Fu Q., Yang X., Li C., Zhou B., Yu Q., Chen L. (2013). Characteristics and ship traffic sources identification of air pollutants in China’s largest port. Atmos. Environ..

[37] Okuda T., Nakao S., Katsuno M., Tanaka S. (2007). Source Identification of nickel in TSP and PM_2.5_ in Tokyo, Japan. Atmos. Environ..

[38] Cao J.J., Lee S.C., Ho K.F., Fung K., Chow J.C. (2006). Characterization of roadside fine particulate carbon and its 8 fraction in Hong Kong. Aerosol Air Qual. Res..

[39] Milando C., Huang L., Batterman S. (2016). Trends in PM_2.5_ emissions, concentrations and apportionments in Detroit and Chicago. Atmos. Environ..

[40] Kundu S., Stone E.A. (2014). Composition and sources of fine particulate matter across urban and rural sites in the Midwestern United States. Environ. Sci. Process. Impacts.

